# Condensation of Exciton–Polaritons in a Bound State in the Continuum: Effects of the Excitation Spot Size and Polariton Transport

**DOI:** 10.1021/acsnano.4c09970

**Published:** 2024-11-09

**Authors:** Anton Matthijs Berghuis, Arjan Boom, Rafael P. Argante, Shunsuke Murai, Jaime Gómez Rivas

**Affiliations:** † Department of Applied Physics and Science Education and Eindhoven Hendrik Casimir Institute, Eindhoven University of Technology, P.O. Box 513, 5600 MB Eindhoven, the Netherlands; ‡ Institute for Complex Molecular Systems-ICMS, Eindhoven University of Technology, P.O. Box 513, 5612 AJ Eindhoven, the Netherlands; § Department of Material Chemistry, Graduate School of Engineering, Kyoto University, Katsura, Nishikyo, 6158510 Kyoto, Japan

**Keywords:** bound states in the continuum, lasing, polariton condensation, metasurface, polariton transport

## Abstract

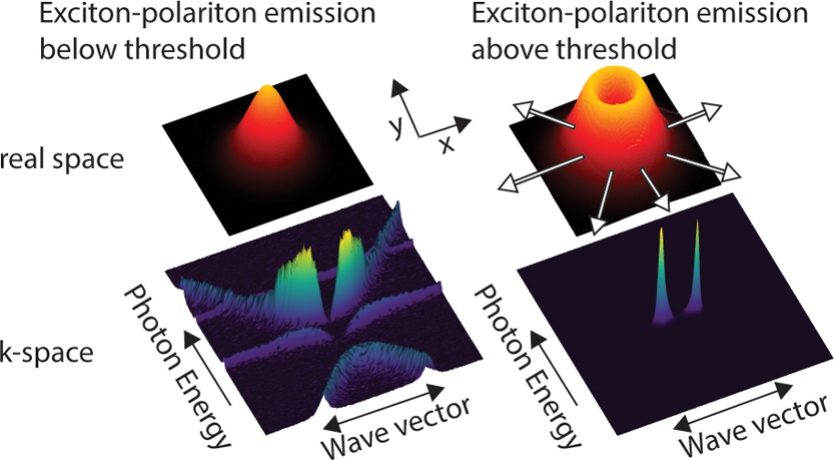

We report the formation of polariton condensates from strongly coupled molecules to bound states in the continuum with quadrupolar character in a metasurface of silicon nanoparticles. Our experiments demonstrate a strong dependence of the condensation threshold on the excitation spot size. The condensation threshold decreases as the excitation spot size increases, achieving thresholds below 3 μm cm^–2^ for spot sizes of around 1 mm^2^ and condensate lifetimes exceeding 20 ps. The strong dependence of the condensation threshold on the spot size is caused by the long propagation length of the polaritons. We reproduce this dependence in simulations by including a term for the ballistic transport of exciton–polaritons in the rate equations describing the condensation. These results illustrate the critical role that polariton transport plays in condensation and highlight the relevance of considering the size of the excitation in condensation experiments.

Bound states in the continuum (BICs) are nonradiative optical modes in structures arising from the symmetry mismatch of the electromagnetic field in the structure and the field of free-space radiation.^1−4^ An example of a structure supporting a BIC is an infinite two-dimensional lattice of Mie scatterers with pure quadrupolar modes.^5−8^ These modes constitute “perfect” cavities with full suppression of radiation leakage and no material losses, serving as an ideal platform for investigating strong light–matter coupling and polariton condensation. If the interaction or energy exchange between excitons and photons is faster than their decay or loss rates, they hybridize, giving rise to new eigenstates called exciton–polaritons (EPs).^9,10^ EPs have partially photonic and excitonic character, resulting in very low effective masses and large nonlinearities.^11^ The low effective mass in combination with the long lifetimes of BICs facilitates the accumulation of substantial polariton populations in a ground state, forming nonequilibrium Bose–Einstein condensates.^12,13^

We have recently demonstrated room-temperature organic polariton lasing from a BIC at low thresholds. This BIC was realized in a system of silicon nanodisks arranged in a square lattice with a period of 420 nm coupled to a layer of perylene dye molecules in a poly(methyl methacrylate) (PMMA) matrix.^8^ While, in theory, it is possible to design a cavity without any losses, in practice, there will be losses originating from the finite size of the lattices, absorption in the nanoparticles, and absorption of the dye molecules. This means that we have to account for both material losses and losses due to the in-plane propagation of EPs escaping from the excited regions in the cavity.^14−16^ Previous studies have shown that polaritons exhibit ballistic propagation with distinct group velocities, efficiently transporting excitations in strongly coupled systems over extremely long distances, reaching up to hundreds of micrometers.^17−22^ Although this property may be promising for applications such as solar cells, where excitons need to propagate to a donor–acceptor interface in which charges are separated,^23−25^ it poses a challenge for the design of low-threshold polariton lasers, where long-range polariton transport can be detrimental.

In this article, we investigate the effect of the excitation spot size on the condensation threshold in a system of a BIC coupled to a layer of perylene dye molecules in a PMMA matrix. We show that in a BIC, where radiation and material losses are suppressed, the in-plane propagation of polaritons dominates the overall polariton losses from the condensate and, therefore, is the main factor determining the polariton lasing threshold. By increasing the excitation spot diameter beyond the ∼400 μm that we used in our previous work, we reduce the threshold by 40% to 3 μJ cm^–2^. Furthermore, the temporal coherence of the condensate decreases for smaller excitation spots from a lifetime of more than 21 ps (corresponding to a *Q*-factor of 66,000) for a large spot to only a few ps for smaller spot sizes. We use a system of coupled rate equations to explain the changes in threshold and temporal coherence. The reduced temporal coherence is evidenced by a spectral broadening of the emission, as well as a shift of the emission to larger wave vectors and photon energies. Our results reveal the importance of the spatial dimension of the optical pump in polariton condensates and emphasize the importance of confining polaritons in nanoscale condensates for applications in low-threshold coherent emission and in quantum information processing.^26−30^

## Results/Discussion

**Figure 1:**
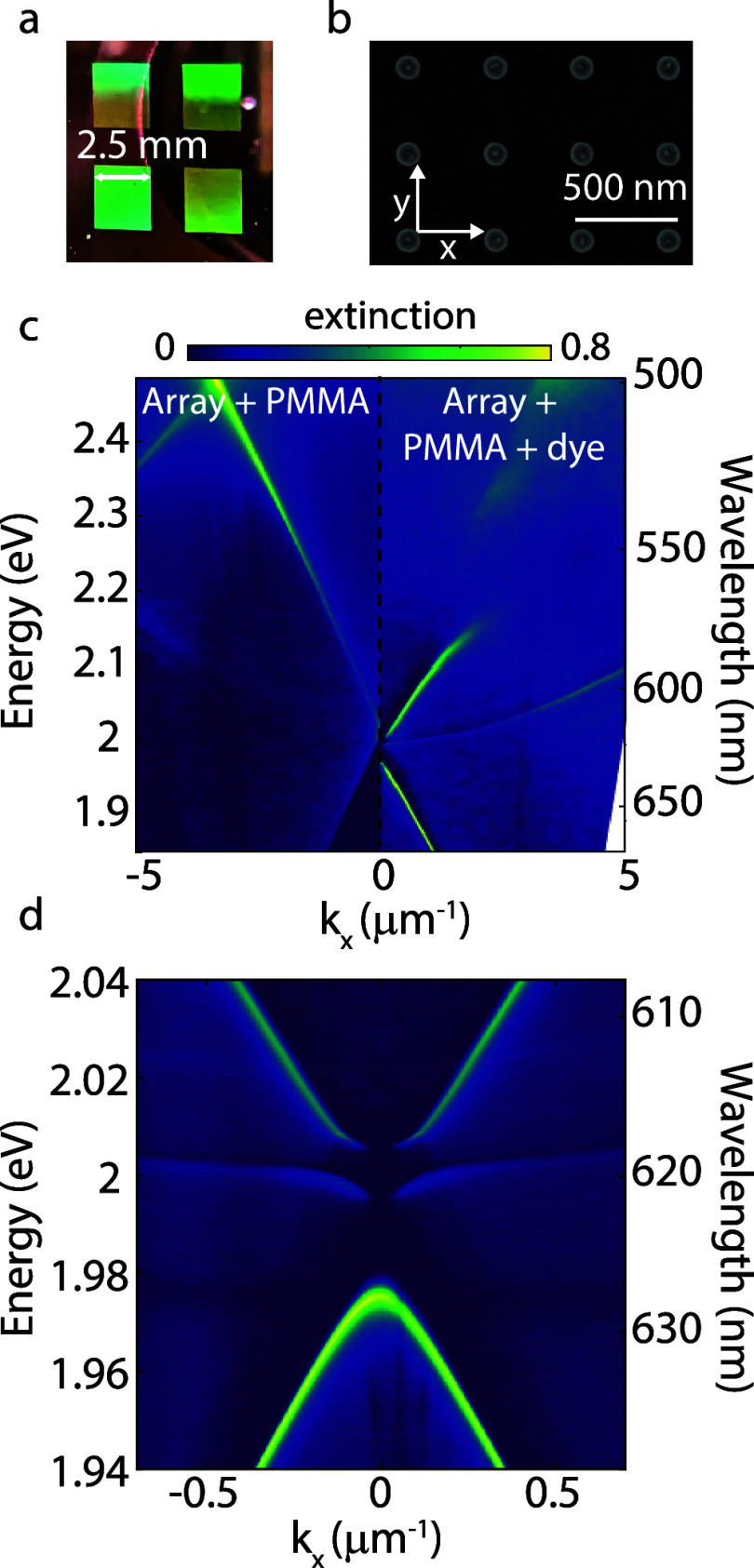
(a) Photograph of the sample. (b) SEM image of the Si nanoparticle array. (c) Left panel: angle-dependent extinction of s-polarized light by the nanoparticle array covered with 200 nm of PMMA. Right panel: extinction of the nanoparticle array with a 200 nm layer of perylene dye in PMMA on top. (d) Close-up view of the extinction for the array covered with the dye film showing two BICs at normal incidence.

The cavity supporting BICs consists of an array of polycrystalline silicon (Si) disks with a height of 90 nm and a diameter of 90 nm on a glass substrate, placed in a square lattice with a unit cell of 420 × 420 nm^2^. This system has been described before in ref (8), and the details of the sample fabrication can be found in the Methods section. Figure 1a shows an optical image of the scattering of white light on four 2.5 × 2.5 mm^2^ arrays, and Figure 1b shows a scanning electron microscopy image of one of the arrays, where the individual nanodisks are visible. We spin-coated a 200 nm layer of PMMA on top of the array and visualized the dispersion of the surface lattice resonances (SLRs) in the bare array under s-polarized light (left panel of Figure 1c). The dispersion is measured by detecting the extinction spectra (1—zeroth order transmittance) as a function of the angle of incidence (θ), defining the in-plane wave vector *k*_*x*_ = (2π/λ) sin (θ). SLRs are the result of the radiative coupling of the individual nanodisks by in-plane diffraction in the array.^31−34^ When we remove the PMMA layer and replace it with a PMMA layer doped with 33 wt% perylene dye, the SLRs couple with the excitons in the molecules at 2.24 and 2.41 eV, as shown by the modified dispersion and the anticrossing seen at 2.24 eV in the right panel of Figure 1c. This anticrossing is characteristic of the formation of EPs in a strongly coupled system.^8^ The resulting EPs have extremely low effective mass due to the large photonic component while allowing EP–EP interactions due to their excitonic component, making this system well-suited for polariton condensation.^11,35−37^ A closer look at the coupled modes around *k_x_* = 0 shows a vanishing extinction for two modes at *k*_*x*_ = *k*_*y*_ = 0 or normal incidence (see Figure 1d). For this wave vector, two BICs are formed, which cannot couple to the incident radiation suppressing the extinction. The BIC at 1.995 eV is associated with a magnetic dipole along the *y*-direction, while the BIC at 2.01 eV corresponds to a quadrupolar mode. The bright mode at normal incidence with an energy of 1.98 eV is an electric dipolar mode. In addition, this structure has a magnetic dipolar mode at 2.02 eV, but this mode is not resolved because of its small extinction cross section. The quality factor of quadrupolar mode diverges as the in-plane wave vector approaches 0, reaching a value of at least 2500, which is the resolution of our spectrometer.^8^

**Figure 2:**
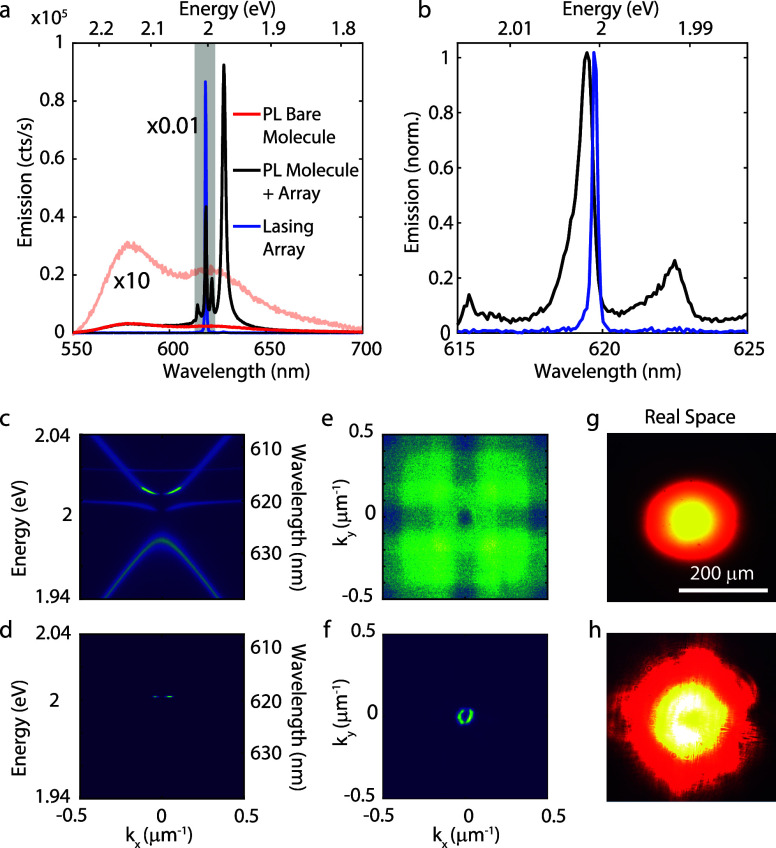
(a) Emission spectra. The red curve shows the fluorescence spectrum of the bare molecule (the pink curve shows the same spectrum ×10). The black curve shows the emission of the perylene molecules on top of the array of Si nanoparticles at an angle of 0.5°. The blue curve shows the emission spectrum of the condensate scaled by a factor of 0.01. (b) Close-up view of the gray area in (a) showing the spontaneous emission (black curve) and condensate emission (blue curve) from the perylene molecules on the array. Angle-resolved PL spectrum from the array with molecules (c) below the threshold and (d) above the threshold is plotted. (e) Emission in the *k_x_*–*k_y_* plane below the threshold using a 620 ± 10 nm bandpass filter and (f) emission above the threshold. Real-space image of the condensate surface is shown (g) below the condensation threshold and (h) above the condensation threshold, showing a very distinct shape.

The bare perylene molecules have broad emission spectra with an electronic transition at 2.16 eV and the first vibronic replica at 2.01 eV, as shown with the red and pink curves in Figure 2a. Note that the pink curve is multiplied by 10 for better visibility. When the dye molecules are on top of the array and for the same excitation conditions, additional sharp peaks arise, which are visible in the emission spectrum. These peaks can be seen at an angle of 0.5° from normal incidence, as shown with the black curve in Figure 2a, and correspond to the four different optical modes described in the previous paragraph. When the pumping fluence increases to ∼5 μJ cm^–2^, the EPs condense into the quadrupolar BIC, and the emission leaks out of this BIC at small angles. This condensation results in an exponential increase in the emission intensity (blue curve in Figure 2a) and the spectral narrowing of the peak, as shown with the blue and black curves in the magnified image in Figure 2b, which results from the enhanced spatial coherence of the condensate.

We investigate the angle dependence of the emission by imaging the back-focal plane in a Fourier microscope using a collection objective with a numerical aperture of 0.3 (10× Nikon CFI Plan Fluor). We excite the sample with the second harmonic of an amplified laser (Astrella Coherent) at 400 nm with a repetition rate of 1 kHz and a pulse duration of ∼100 fs. The emission of the dye on top of the array below the threshold for condensation, as plotted in Figure 2c, follows the same dispersion as the extinction measurement shown in Figure 1d. The magnetic dipolar mode along the *x*-direction that was not resolved in Figure 1d is now visible at 2.02 eV due to the better signal-to-noise ratio of the emission measurement compared to the transmission measurement. By imaging the Fourier plane using a 620 ± 10 nm bandpass filter, the C_4_ symmetry of the quadrupolar modes in the square array is visible as a function of *k*_*x*_ vs *k*_*y*_, as plotted in Figure 2e. The intensity of the mode vanishes at *k_x_* = *k_y_* = 0, as expected for a BIC.

**Figure 3:**
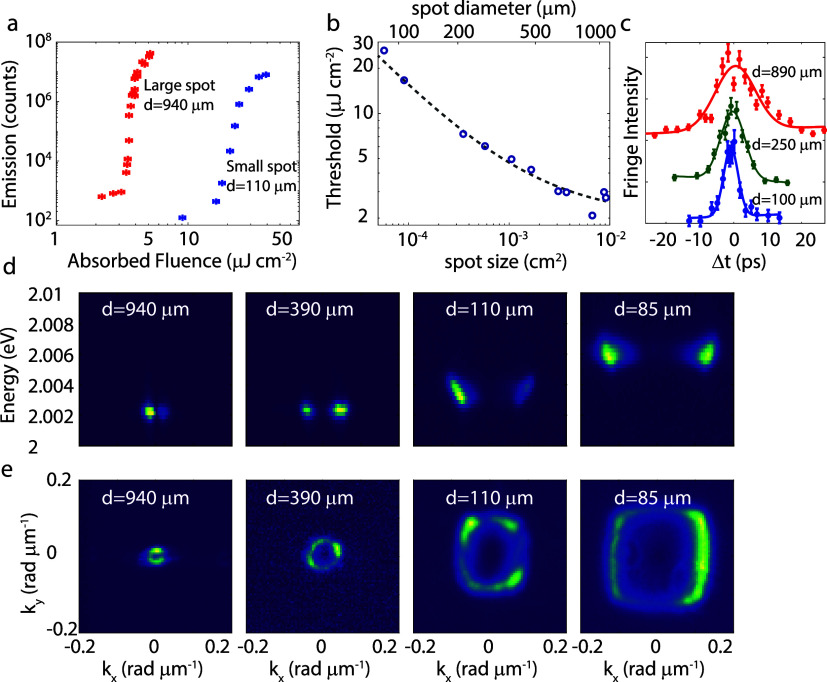
(a) Threshold measurements for a large excitation spot (blue crosses) and a small excitation spot (red crosses). (b) Condensation threshold as a function of the excitation spot size. The dotted curve is an exponential fit to the measurements with the equation *P*_th_ = 2.03 + 0.02 · *F*^–0.7^, with *F* being the spot size in cm^2^. (c) Intensity of the interference fringes in the *g*^(1)^ correlation measurements as a function of the time delay between the overlapped images for three different excitation spot sizes. (d) Momentum–energy relations for different spot sizes, illustrating the blue shift and the emission at larger wave vectors for smaller excitation spots. (e) Emission from the condensates in momentum space for different spot sizes.

The condensation of EPs is evidenced above the threshold by the emission originating from a defined energy (2.01 eV, Figure 2d) and wave vector (0.03 μm^–1^, Figure 2f), with a typical torus shape, evidencing the topological character of the BIC.^38^ The slight asymmetry in the emission profile above the threshold in Figure 2f is due to inhomogeneities in the sample or an asymmetry of the laser excitation. When we image the surface of the nanoparticle array, we can see that below the threshold, the emission has a nearly Gaussian shape, which corresponds to the shape of the excitation laser beam (Figure 2g). Above the threshold, however, the emission no longer follows the excitation profile but instead has a torus shape with lower intensity in the center.^39−41^ Interestingly, we found that the area of emission increases beyond the size of the excitation beam. In the absence of EP transport, we would expect a reduction of the emission area since the polariton condensation has a nonlinear dependence on the excitation density. The fact that the emission area above the threshold is significantly larger suggests that EP transport plays a significant role in the dynamics of the condensate. To validate this hypothesis, we measured the condensation threshold for different sizes of the excitation spot and measured the emission maximum as a function of the total absorbed fluence of the laser by the perylene-doped PMMA layer on the array. A threshold measurement for a large excitation spot with a full width at half-maximum (fwhm) of 940 μm and a small excitation spot with an fwhm of 110 μm is plotted with the blue and red crosses in Figure 3a. In these measurements, the condensation threshold is characterized by the nonlinear increase of the emission. By decreasing the size of the excitation spot from *d* = 940 to *d* = 110 μm, the threshold increases from ∼3.1 to almost 20 μJ cm^–2^. While the influence of the excitation spot size can have a greater impact on the condensation threshold than the intrinsic properties of the optical cavity enabling strong coupling, the spot sizes are often not reported in the literature, which makes comparison of the performance of different systems impossible. To study this behavior more systematically, we have plotted the threshold for several different excitation spot sizes with the blue circles in Figure 3b. The gray dotted curve is an exponential fit to the data (*P*_th_ = 2.03 + 0.02 · *F*^0.7^, with *F* being the spot size in cm^2^), suggesting that the minimum condensation threshold for a very large spot from this BIC is approximately 2 μJ cm^–2^. When the illuminated area becomes larger than ∼1 mm^2^, the losses are no longer limited by the size of the excitation spot but by the material losses in the Si nanoparticles and the nonradiative losses in the dye molecules.

To study the effects of the size of the excitation beam in more detail, the temporal coherence of the condensate was determined by overlapping the image of the polariton emission with its mirror image in a Michelson interferometer and measuring the amplitude–amplitude or first-order correlation function (*g*^(1)^). The position of the retroreflector in the interferometer was scanned to change the time delay (Δ*t*) between the two images (the images measured in this setup can be found in SI Section S1). From the visibility of the interference fringes as a function of Δ*t*, as shown in Figure 3c, the coherence time (*t*_coh_) can be calculated as , where sigma is the variance of the Gaussian fits shown with solid curves in Figure 3c. The measured coherence times are *t*_coh_ = 6.1, 13.1, and 21.8 ps for spot sizes with diameters of 100, 250, and 890 μm, respectively. The coherence time of the largest spot can be associated with a *Q* factor of 66,000 (*Q* = 2πν*t*_coh_), which is a lower bound for the actual lifetime of the cavity.^42^ This lifetime and the corresponding group velocity at the wave vector of condensation (*k* = 0.03 rad μm^–1^) results in propagation length of the polaritons of up to 210 μm for the largest spot size (see SI section S3 for the calculation). The decreasing coherence time at a smaller excitation spot size could be caused by the higher density of EPs required to reach condensation, which results in EP–EP interactions and decoherence.

We also systematically analyzed the energy and wave vector of the condensate along the *x*-direction (*k*_*x*_) by varying the excitation spot size in a Fourier microscope. Our observations reveal a clear trend: as the spot size decreases, the energy of the condensate increases, as well as showing a broadening of the emission (see Figure 3d). The blue shift of the emission is accompanied by a shift toward larger wave vectors, as illustrated in Figure 3d. The broadening is directly related to the shorter coherence time of the condensate, but the blue shift of the mode needs further explanation. It should be noted that the blue shift cannot be explained by the increasing density of the polaritons alone, as this effect is much smaller (as we have shown in SI Section S2). Instead, the blue shift can be attributed to the more efficient radiative relaxation from the reservoir excitons into the cavity mode at higher energies compared to the BIC, which becomes an important factor for the threshold value when the losses are limited, as will be discussed in more detail with eqs 1 and 2. Notably, as the emission extends to larger angles, the torus shape transforms into a more square-like emission on k-space. This intriguing pattern finds a straightforward explanation in the distinct shapes assumed by the optical modes at higher energies, as depicted in SI Section S4.

**Figure 4:**
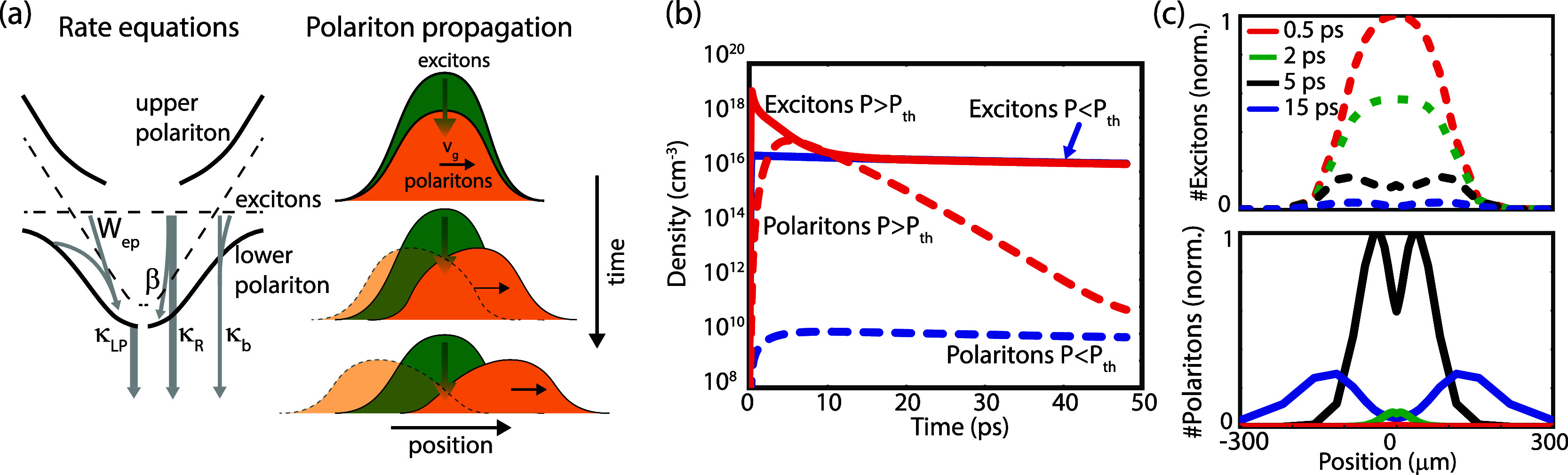
(a) Illustration of the kinetic model indicating the different decay rates. The image on the right shows the influence of implementing a group velocity for the polaritons, where the upper image corresponds to the exciton and polariton populations shortly after excitation and the two lower images are the populations at later times. (b) Simulated time evolution of exciton and polariton densities at the center of the excitation spot for a spot size of σ = 50 μm. The solid blue curve and dashed blue curve show the exciton and polariton densities for an initial exciton density of 0.4 μJ cm^–2^ (below the threshold), while the red solid and dashed curves show the exciton and polariton densities for an initial excitation energy of 80 μJ cm^–2^ (above the threshold). (c) Spatial distributions of the excitons (top) and polaritons (bottom) above the threshold for different times after excitation.

To explain the decreasing threshold and other optical properties of the condensate qualitatively, we use a kinetic model describing the time evolution of the exciton reservoir (*n*_R_) and the lower polariton (*n*_LP_),^30,43−45^ including the ballistic propagation of EPs away from the excitation spot^41,46^
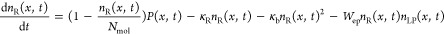
1
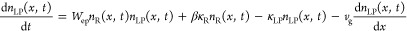
2Here, *N*_mol_ is the total number of dye molecules and *P*(*x*,*t*) is the temporal and spatial shape of the excitation beam. We illustrate the other variables of the model with the two figures in Figure 4a. The left panel shows the rates between the exciton reservoir and the lower polariton and the decay to the ground state. κ_R_ is the decay rate of the reservoir excitons, and a fraction β of the decayed excitons populates the polaritons (analogous to the spontaneous emission factor in conventional lasers). κ_b_ is the bimolecular recombination rate, and κ_LP_ is the EP decay rate. *W*_ep_ is the EP scattering rate representing the bosonic final state stimulation. This is a nonlinear term that depends on both the number of exciton states and (final) polariton states and is the driving mechanism for condensation.^45^ To account for the propagation of EPs, we include a spatial variable (*x*) and a term for ballistic transport^47−51^ in eq 2 involving a constant group velocity *v*_g_ and the gradient of the number of polariton states , while we omit the slow diffusion contribution to the propagation. We illustrate this in the right panel of Figure 4a. While the polaritons propagate away from the center of the spot, the exciton reservoir is stationary and keeps populating the polaritons at the center. Since the transport is ballistic, we can model the polaritons traveling in the negative direction independently from those traveling in the positive directions. This ballistic transport is also clear from our experimental results, as diffusive transport could not result in the torus-shaped emission profile shown in Figure 2h.

We numerically evaluate the rate equations for a spot with an initial size of σ = 50 μm and a low excitation density of 0.4 μJ cm^–2^ (below the threshold). The number of exciton and polariton states at the center of the excitation spot is plotted with the blue solid and dashed curves in Figure 4b. At these low excitation densities, the role of *W*_ep_ is only small, and the excitons and polaritons quickly reach a quasi-equilibrium. When the excitation energy density increases to 80 μJ cm^–2^, the nonlinear EP scattering becomes dominant and results in a very rapid increase of the polariton density enabling condensation, as is shown with the red solid and dashed curves in Figure 4b. We can also investigate the spatial distribution of the excitons and polaritons above the threshold, as shown in Figure 4c. The red dashed curve in the top panel shows the initial distributions of excitons with a Gaussian shape just after excitation. At this time, there are barely any polaritons in the excited state, as shown by the solid red curve in the bottom panel. The polariton population rapidly grows while the exciton density decreases (especially in the center of the excitation spot, where the exciton density is the highest), as shown with the green dashed and solid curves. The growth continues during the first 5 ps after excitation, and the transport of the polaritons away from the excitation spot becomes clear, resulting in a dip of the polariton density in the center, as shown with the black curves. As time proceeds, the polaritons continue to propagate and decay to the ground state, as shown with the blue dashed and solid curves in Figure 4c.

In Supporting Information Section S7, we study the dependence of the condensation threshold on the variables *W*_ep_, κ_LP_, and β. We find that the threshold scales by *W*_ep_ according to a power law (the threshold reduces by a factor of n when *W*_ep_ is increased by a factor of n). There is a similar relationship between κ_LP_ and the threshold, as long as κ_LP_ is much smaller than κ_R_ (i.e., the threshold reduces by the same factor n when κ_LP_ is reduced by a factor of n). However, the threshold is almost independent of β. Since the influence of β on the condensation threshold is much smaller than the influence of *W*_ep_ and the cavity decay rate κ_LP_, the polaritons will generally form the condensate in a mode with a longer lifetime instead of a mode with a larger β factor. However, when the *Q*-factor of the BIC is limited by the size of the excitation spot as a result of the EP propagation away from the excitation area, the longer intrinsic lifetime of the BIC is no longer an advantage for reaching a low condensation threshold. For small excitation spots, condensation into a mode with a larger β-factor may therefore have a lower threshold, which is in agreement with the observation in Figure 3d, where smaller excitation spots resulted in condensation into a mode with a larger β-factor (lower *Q*-factor). These modes with larger β-factors are at larger angles, as confirmed by the simulations in SI Section S4.

**Figure 5:**
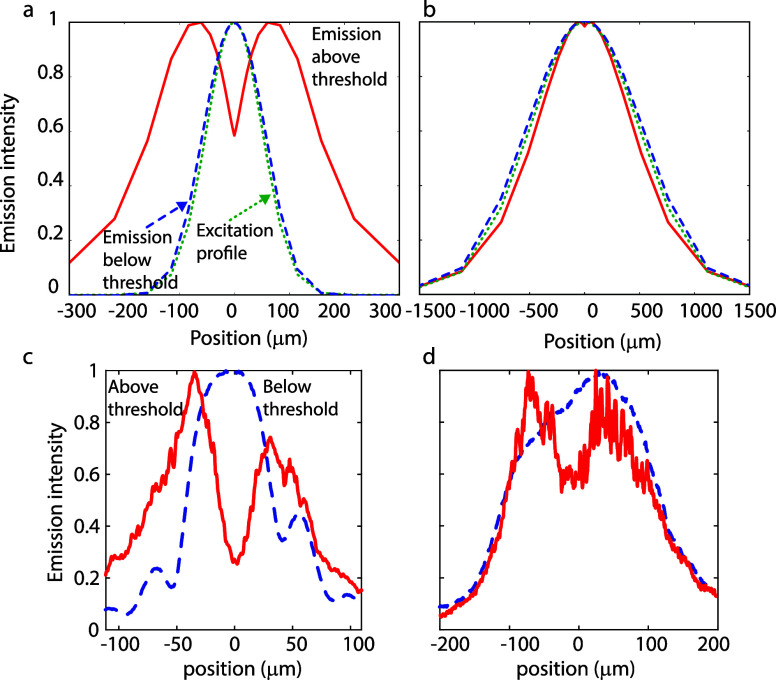
Simulated emission profiles below (blue dashed curves) and above (red curves) the condensation threshold for a small (a) and a large (b) excitation spot (green dashed–dotted curves). The parameters for the simulation are given in Table 1. (c) and (d) Measured emission profiles below and above the threshold for small and large excitation spots, respectively.

To qualitatively understand the experimental observations of the condensation, we numerically solve the rate equations with a 1D Gaussian excitation profile with different variances (fwhm ∼2.355 σ) and a time step of 0.4 fs. Since our experiments measure the time-integrated exciton and polariton distributions, we also plot the time-integrated exciton and polariton densities. The results using the parameters given in Table 1 are presented in Figure 5. At low excitation densities, EPs only make up a small fraction of the total number of excited states, and the emission profile closely resembles the excitation spot, as indicated with the blue dashed and green dotted curves in Figure 5a. Above the threshold, the EP distribution dominates the emission profile as a result of the shorter radiative lifetime of the polaritons compared to that of the reservoir states. Initially, EPs are formed at regions with the highest exciton reservoir concentration, but as the polaritons propagate toward the edge of the excitation profile, their population builds up due to the continuous stimulated scattering of reservoir states to the lower polariton band. Therefore, the integrated emission profile for an excitation spot of 120 μm has its maxima away from the center of the excitation spot, as shown with the solid red curve in Figure 5a (a more detailed analysis is given in SI Sections S5 and S6). The simulated behavior of the emission profile is in qualitative agreement with the experimentally observed emission profiles above and below the threshold, as shown by the blue dashed and solid red curves in Figure 5c. When the excitation spot size is increased, the emission profile of the condensed EPs looks very different. The relative influence of the EP propagation away from the excitation area is much smaller. Because the condensation is more efficient at the center of the excitation spot due to the higher number of excitons and the nonlinear EP scattering (eqs 1 and 2), the emission profile above the threshold is even smaller than the initial excitation. The simulation qualitatively agrees with our experiments in Figure 5d; however, it is clear that the intensity reduction at the center of the excitation spot is more pronounced in the experiment. This discrepancy could be caused by the simplifications in the model (note that we used a 1D model while the condensation occurs on a surface) and an underestimation of the group velocity of the polaritons. It is however interesting that the ballistic transport of the polaritons, in combination with the nonlinear buildup of the polaritons, is sufficient to reproduce the hole burning without any term of polariton–polariton repulsion.

**Table 1:** Simulation Parameters used for Modeling Polariton Condensation

parameter	value
*N*_mol_	2.7 × 10^20^ cm^–3^
κ_R_	1.6 × 10^–3^ ps^–1^
κ_LP_	0.125 ps^–1^
κ_b_	0.5 × 10^–18^ cm^3^ ps^–1^
*W*_ep_	2.6 × 10^–18^ cm^3^ ps^–1^
β	5 × 10^–5^
*v*_g_	10^7^ m s^–1^

**Figure 6:**
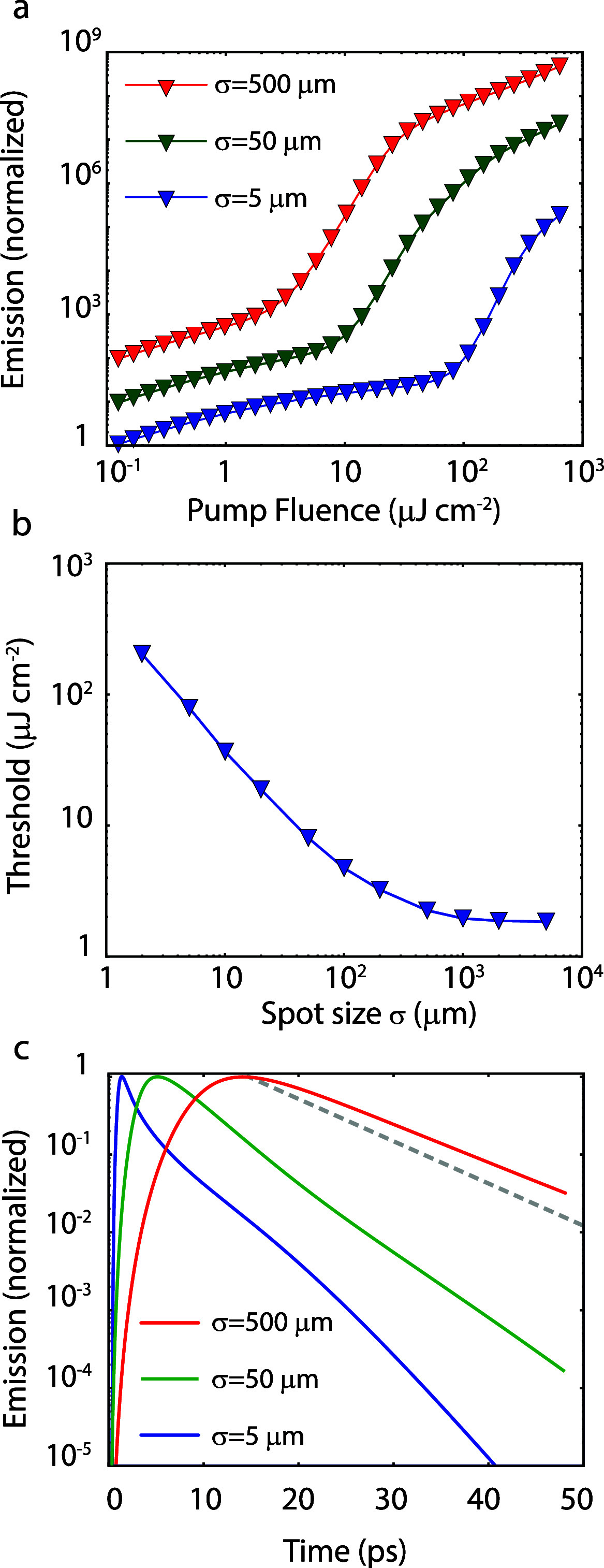
(a) Simulated threshold curve for three spot sizes. The curves show the sum of the emission from the exciton reservoir and the polariton states and are normalized to the total emission from a 5 μm spot excited at a fluence of 0.125 μJ cm^–2^. (b) Condensation threshold calculated for different excitation spot sizes. The solid curve is a guide to the eye. (c) Time-resolved emission intensity above the condensation threshold for three different excitation spot sizes integrated over the area of the excitation. The gray dashed curve shows the emission decay corresponding to the intrinsic EP lifetime without considering EP propagation or population from the reservoir.

The simulated EP distribution in Figure 5 explains the strong dependence of the polariton threshold on the excitation spot area since the propagation of polaritons out of the exciton reservoir region is an additional loss channel of EPs. To quantify this effect, the simulated threshold curves for three different spot sizes are shown in Figure 6a. From these and more simulations, the condensation threshold is determined and plotted in Figure 6b, showing the reduction of the threshold with increasing excitation spot size until this spot is large enough such that the spatial dimensions are no longer the limiting factor of the lifetime of the polaritons. This simulated threshold dependence closely resembles the experimental threshold curve plotted in Figure 3b. The associated lifetimes of the condensates formed with different excitation spot sizes are plotted in Figure 6c, showing indeed a reduction of the condensate lifetime for smaller excitation spots at the threshold.

## Conclusions

In conclusion, we have shown experimentally the reduction of the condensation threshold of EPs in a bound state in the continuum by increasing the excitation spot size. This dependence is modeled using coupled rate equations for the exciton reservoir and the lower polariton and incorporating the ballistic propagation of the EPs. Furthermore, we have shown that for small spot sizes, the condensation takes place at larger wave vectors and energies. This phenomenon is explained because condensation is a trade-off between the lifetime of the polaritons and the population rate of the lower polariton from the exciton reservoir states. This strong dependence of the condensation threshold on the excitation spot size, especially in low-loss cavities, makes it essential to report the spot size when measuring the condensation threshold. Furthermore, these results illustrate that to make nanoscale condensates, it is important to confine the EPs in optical modes with low group velocities while still maintaining a long lifetime.

## Methods/Experimental Section

The sample fabrication is described in ref (8). Polycrystalline Si films with a thickness of 90 nm were grown on a synthetic silica glass substrate by low-pressure chemical vapor deposition using SiH_4_ gas as a source of Si. A resist (NEB22A2, Sumitomo) was cast on the Si film and exposed to electron-beam lithography, followed by development to make nanoparticle arrays of resists on the Si film. The Si film was vertically etched using selective dry etching (Bosch process) with SF_6_ and C_4_F_8_ gases, and the resist residue was etched away by dry oxygen etching. The fabricated array covered an area of 2.5 × 2.5 mm^2^. After the fabrication of the metasurface, a solution of 32 wt% perylene dye ([*N*,*N*′-bis(2,6-diisopropylphenyl)-1,7- and -1,6-bis(2,6-diisopropylphenoxy)-perylene-3,4:9,10-tetracarboximide]) in PMMA was spin-coated to form a 230 nm thick layer.

## Supporting Information Available

Coherence time measurements (S1); effect of the pump fluence on the emission (S2); propagation length from polariton dispersion (S3); rigorous coupled-wave analysis (RCWA) of the BIC mode (S4); simulated time-resolved polariton condensation (S5); simulated spatially resolved polariton condensation (S6); and threshold dependence on simulation variables (S7) (PDF)
