# Supplementary material for: Condensation of Exciton–Polaritons in a Bound State in the Continuum: Effects of the Excitation Spot Size and Polariton Transport
Source: ACS Nano. 2024 Nov 9;18(46):31987–94. doi: 10.1021/acsnano.4c09970 (PMC11580381; doi:10.1021/acsnano.4c09970)
Supplement: Supplementary file 1 — nn4c09970_si_001.pdf [file nn4c09970_si_001.pdf]

Supporting Information for "Condensation of Exciton-Polaritons in a Bound State in the Continuum: Effects of the Excitation Spot Size and Polariton Transport"

Anton Matthijs Berghuis[1,2], Arjan Boom[1,2], Rafael P. Argante[1,2], Shunsuke Murai[3], and Jaime Gómez Rivas[1,2]

[1]Department of Applied Physics and Science Education, and Eindhoven Hendrik Casimir Institute, Eindhoven University of Technology,, P.O. Box 513, 5600 MB Eindhoven, The Netherlands.
[2]Institute for Complex Molecular Systems-ICMS, Eindhoven University of Technology, P.O. Box 513, 5612 AJ, Eindhoven, The Netherlands
[3]Department of Material Chemistry, Graduate School of Engineering, Kyoto University, Katsura, Nishikyo, 6158510, Kyoto, Japan.

Contents

S1 Coherence Time Measurements **S2**

S2 Effect of the Pump Fluence on the Emission **S3**

S3 Propagation Length from Polariton Dispersion **S3**

S4 Rigurous Coupled Wave Analysis (RCWA) of the BIC Mode **S5**

S5 Simulated Time-Resolved Polariton Condensation **S6**

S6 Simulated Spatial-Resolved Polariton Condensation **S7**

S7 Threshold Dependence on Simulation Variables **S8**

S1 Coherence Time Measurements

The temporal coherence of the condensate was measured by overlapping the image of the polariton emission with its mirror image on a Michelson interferometer. The position of the retro-reflector was changed to vary the time delay between the two images, resulting in the $g^{(1)}$ correlation image, as shown in Fig. S1 (a) for a spot size of 250 µm. From the visibility of the fringes (Fig. S1 (b)), the coherence time can be calculated and is found to depend on the size of the excitation spot. The obtained coherence times are 6.1, 13.1 and 21.8 ps for spot sizes with a diameter of 100 µm, 250 µm, and 890 µm, respectively.

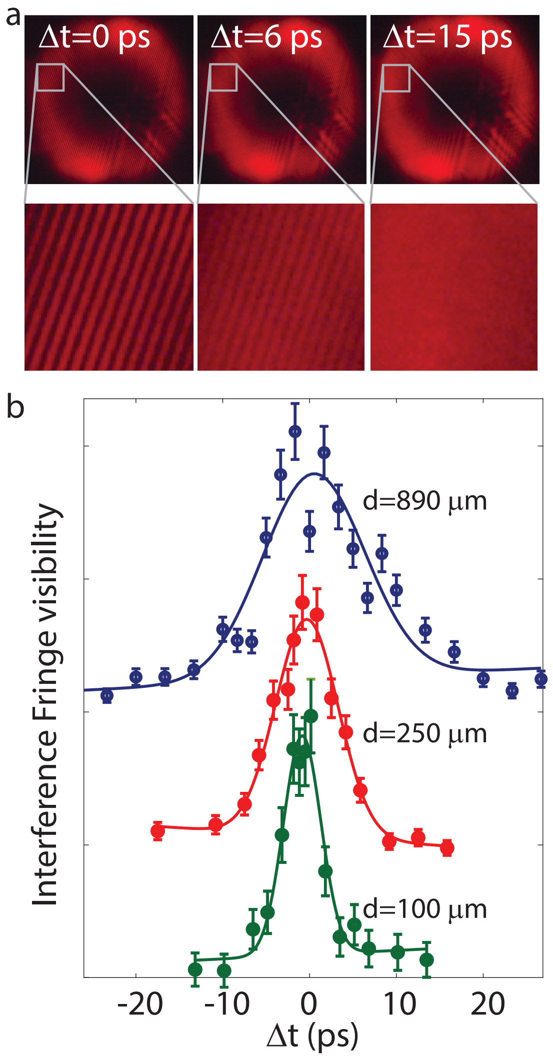

Figure S1: (a) $g^{(1)}$ correlation images of the emission from the polariton condensate for different time delays. The lower row displays magnifications of the image to show the interference fringes. (b) Intensity of the interference fringes as a function of the time delay between the overlapped images for three different excitation spot sizes. The solid curves are Gaussian fits to the measurements from which the coherence time is derived.

S2 Effect of the Pump Fluence on the Emission

To verify that the origin of the modified angular emission from the condensate is due to the change in spot size and not just due to the higher fluence, we have measured the polariton emission at different fluences above the threshold, as shown in Figs. S2 (a-d). The increase in fluence only slightly changes the angular distribution of the emission. When a smaller area is excited with a similar energy per area of 24 μ J cm^{-2}, as in Fig. S2 (e), the distribution of the emission is completely different, confirming that the dimensions of the excitation spot are the dominant factor determining this emission.

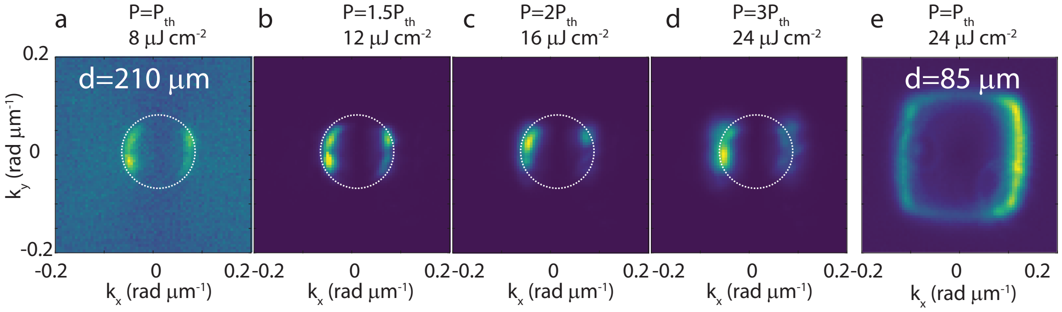

Figure S2: (a-d) Condensate emission in reciprocal space for an excitation spot with a diameter of ∼210 μm at different power densities above the threshold. (e) Polariton condensate emission for a similar fluence as (d), but for a smaller excitation spot size of d=85 μm .

S3 Propagation Length from Polariton Dispersion

The propagation length of the polaritons can be determined form the lifetime of the polaritons and the group velocity. The lifetime is obtained from the coherence time measurements shown in SI section S1. For the largest spot size with d=890 μm, the coherence time is 21 ps. We obtain the group velocity from the polariton dispersion from the derivative of the dispersion ($v_g = \frac{d\omega}{dk}$) as shown in Fig. S3 a and b. The slope to the dispersion at the emission wave vector of the condensate as shown for (k=0.03μm^{-1}) is plotted in Fig. S3 (a) and corresponds to a group velocity of 10^7 ms^{-1}. Therefore, for the largest spot size (d=890 μm) the polariton propagation length is approximately 210 μm.

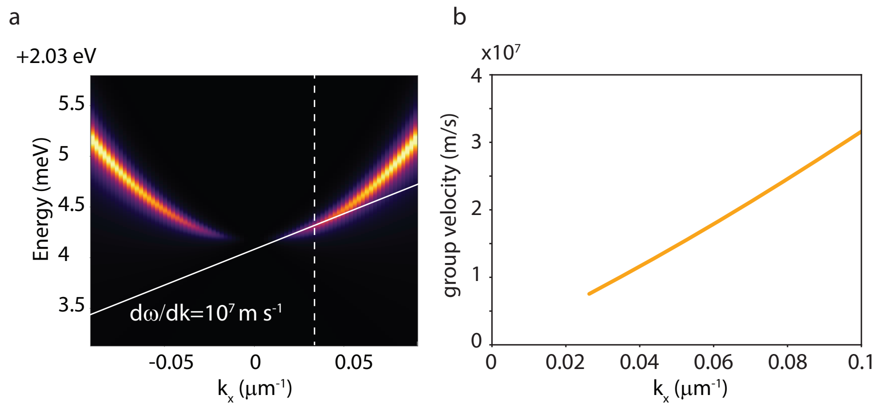

Figure S3: (a) Simulated dispersion of the lower polariton around normal incidence along with the tangent at k=0.03 rad μm^{-1} corresponding to the condensation wave vector for the excitation spot of 890 μm. The the slope of this line is defines the group velocity; $v_g = \frac{d\omega}{dk} = 10^7$ ms^{-1}. (b) The group velocity as a function of the in plane wave vector obtained from the derivative of the dispersion.

S4 Rigurous Coupled Wave Analysis (RCWA) of the BIC Mode

The optical extinction of the metasurface is simulated using the RCWA method. The metasurface consists of Si nanodisks with a height of 90 nm and diameter of 90 nm, placed in a lattice with a period of 420 nm. The particles are covered with a 210 nm thick layer of PMMA mixed with the perylene dye (the permittivity of this layer was determined by ellisopmetry). The resulting angular dispersion is plotted in Fig. S4 (a), while a more detailed simulation of the BIC mode around k=0 is shown in Fig. S4 (b). Although the Q-factor increases for angles closer to normal incidence, the extinction also decreases at smaller angles, as shown in Fig. S4 (c). To obtain the modes as a function of k_x and k_y, the extinction is plotted at several fixed photon energies in Fig. S4 (d). It is clear from this figure that at higher energies, the mode profile in k-space becomes more square-like, which agrees excellently with the experimental angular distribution of the emission of the polariton condensate for small spot sizes.

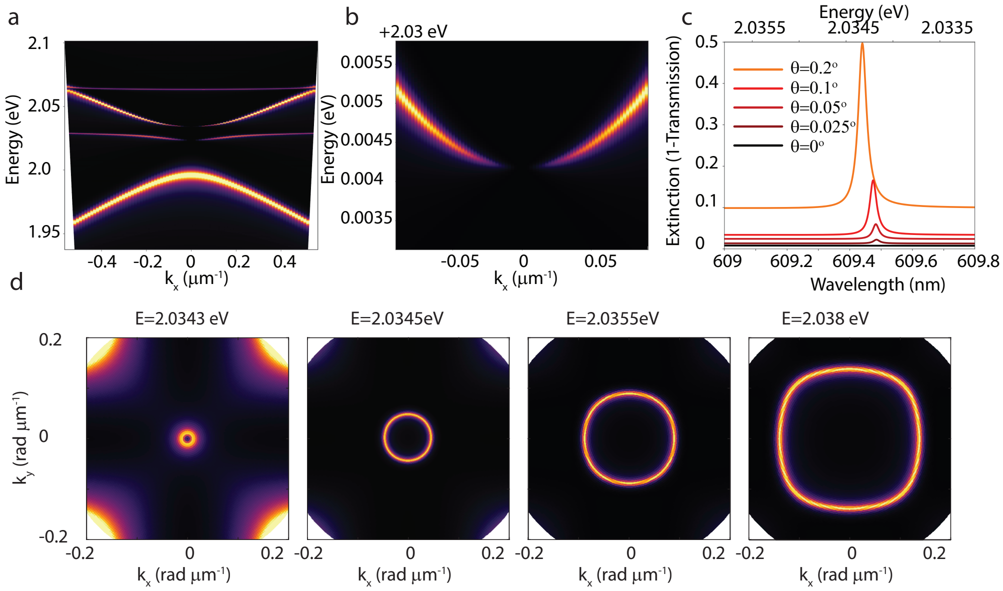

Figure S4: (a) Simulated dispersion of the extinction of the metasurface for s-polarized light. (b) Magnification of (a). (c) Extinction spectra of the mode for different small angles. (d) Extinction in reciprocal space for different energies.

S5 Simulated Time-Resolved Polariton Condensation

In the process of polariton condensation, reservoir excitons decay to the bottom of the polariton band. A polarition condensate is a non-equilibrium Bose-Einstein condensate, as a result of the decay of the polaritons. As discussed in the manuscript, the dominant decay mechanism for a BIC is the propagation of the polaritons from the excitation area. In Fig. S5, we show the simulations of the different populations within the excitation area for spot sizes of 5, 50, and 500 µm. The blue curves display distributions below the threshold at an excitation density of 0.4 μJcm^{-2}, showing that the exciton concentration for all spot sizes is the same below the threshold. However, polariton concentrations (indicated with the blue dashed curves) reach higher densities for the larger spot sizes because of the relatively smaller losses resulting from polariton propagation. Above threshold, the differences are even larger, as shown by the red curves. These curves are simulated at the fluence where the polariton emission saturates, which corresponds to 470 μJcm^{-2}, 80 μJcm^{-2} and 33 μJcm^{-2} for spot sizes of 5, 50, and 500 µm, respectively. In all cases there is a rapid transition from reservoir excitons (solid red curves) to the polaritons (dashed-red curves) but for a smaller spot size, this transition needs to be even faster, because of increased propagation losses. Therefore, the maximum number of polariton states is reached sooner after excitation for smaller spot sizes, but the polaritons in the small spot also decay much faster.

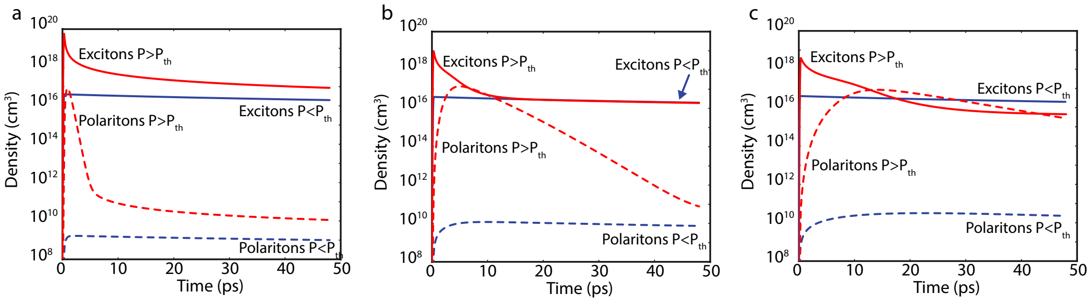

Figure S5: Exciton and polariton densities at the excited area as a function of time for different excitation sizes. (a) Densities for a spot size of $\sigma = 5$ µm, (b) for a spot size of $\sigma = 50$ µm, and (c) for a spot size of $\sigma = 500$ µm The solid-blue curves and the dashed-blue curves show the exciton and polariton densities below threshold, respectively. The polariton densities above threshold are plotted with the dashed-red curves, and the exciton densities above threshold are plotted with the solid-red curves.

S6 Simulated Spatial-Resolved Polariton Condensation

When polaritons are excited in a small region of the sample, they will ballistically propagate away from this region with the group velocity of the polaritons. Therefore, it is intuitive that at a certain time after excitation, the polariton distribution will have a torus-like shape. It is however not immediately clear why the integrated emission from these polaritons has a torus-like distribution as well. To better understand the formation of the polariton condensate and the torus-like emission, we plot the polariton and exciton distributions for different times after excitation and for spot sizes of 5, 50, and 500 µm in Fig. S6. Although initially the polaritons are generated at the center of the excitation spot, their population quickly builds up as they propagate from the center because of the (nonlinear) stimulated scattering of reservoir states to the lower polariton band. Because the polaritons have moved away from the excitation spot, the stimulated scattering is slower in the center of this spot at later times, explaining the torus shape of the emission. For larger spots this effect is much less pronounced and only a small emission intensity dip is visible at the center.

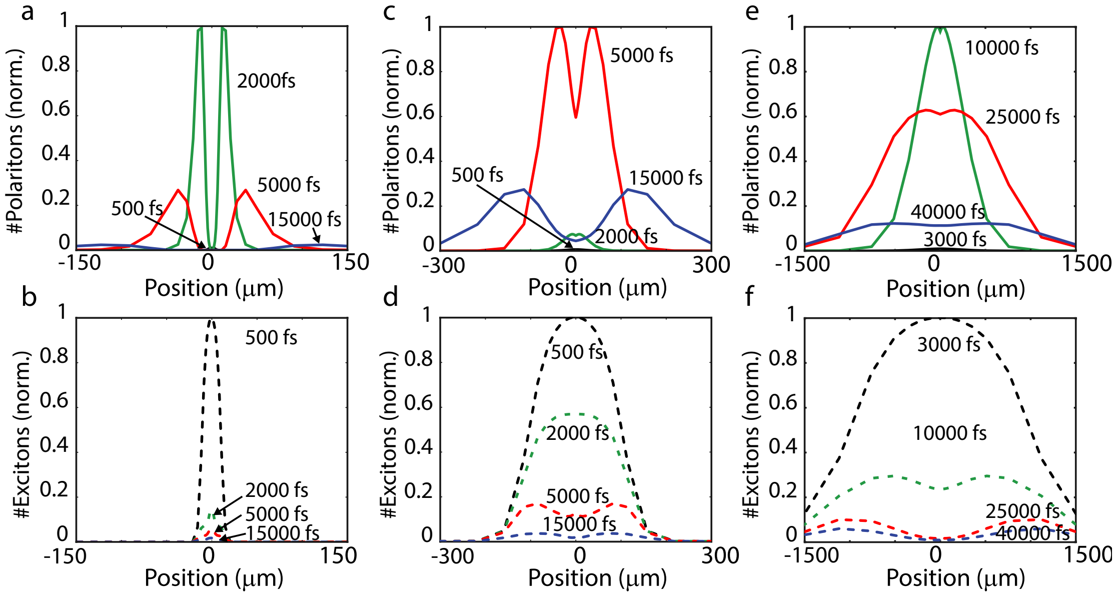

Figure S6: The exciton and polariton spatial distributions for different times after excitation for a spot size of $\sigma = 5$ µm (a and b), $\sigma = 50$ µm (c and d), and $\sigma = 500$ µm 5 (e and f). (a,c and e) show the number of polariton states and (b,d and f) the number of reservoir excitons.

S7 Threshold Dependence on Simulation Variables

In Fig. S7 we simulated the dependence of the condensation threshold as a function of the variables W_{ep}, κ_{LP} and β used in Eqs. 1 and 2 in the main text. In the figure the integrated number of polariton states over the first 10 ns ($\int N_{pol}(t)dt$) is plotted in the color maps as a function of the different variables and the number of absorbed photons. The threshold is determined as the excitation density where the second derivative of the number of polariton states with respect to the excitation intensity (P) becomes larger than 0 on a logarithmic scale i.e. $\frac{d^2 log_{10}(\int N_{pol}}{dP^2})$. The threshold is plotted with the solid red curves in Fig. S7 a-c. From the figure, we can see that the threshold has a power law dependence on W_{ep}, i.e. when the exciton polariton scattering rate is increased by a factor 10, the threshold will decrease by a factor 10. There is a similar dependence of the threshold on the polariton relaxation rate (κ_{LP}), as is shown in panel b. When the relaxation rate is 10 times slower, the threshold becomes 10 times lower as long as the exciton reservoir decay rate (κ_R) is much slower than the polariton relaxation rate. When the κ_{LP} approaches κ_R (indicated with the vertical red dashed line in Fig. b), the threshold becomes limited by the relaxation rate of the reservoir polaritons. In contrast, the condensation threshold is almost independent of the value of β, as shown in Fig. c. The dark red dashed curves in Figs. a-c indicate the point where the number of polariton states N_{pol} reached as value of 10^{16}. For simplicity, the bi-molecular exciton recombination rate is set at 0 for these calculations.

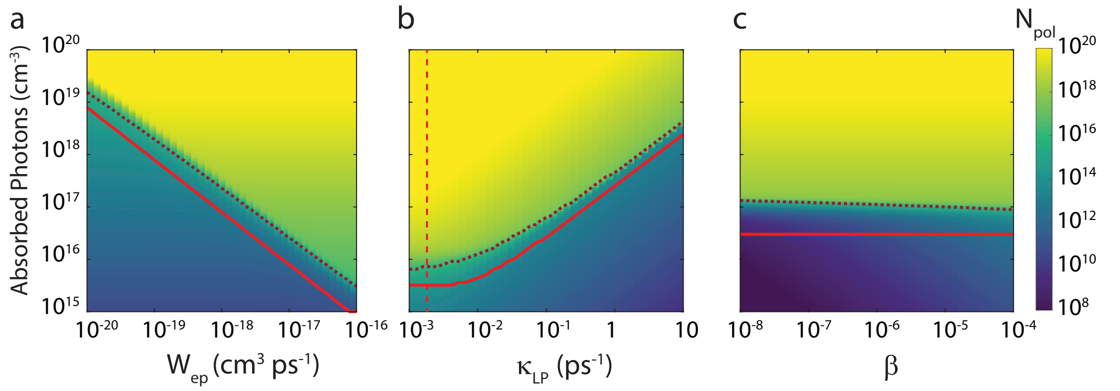

Figure S7: The colormaps show the integrated number of polariton states during the first 10 ns after excitation. (a) shows the dependence of the threshold on exciton-polariton scattering rate (W_{ep}) (b) the dependence on the polariton decay rate (κ_{LP}) and (c) the dependence on the spontaneous emission factor β. The red solid curves in Figs. (a-c) indicate the condensation threshold, the red dashed curves indicate the point where the number of polariton states N_{pol} reached as value of 10^{16}. The vertical red dashed line in (b) indicates the value where κ_{LP}=κ_r.
